# How much does the human medial gastrocnemius muscle contribute to ankle torques outside the sagittal plane?^☆^

**DOI:** 10.1016/j.humov.2013.03.003

**Published:** 2013-08

**Authors:** Taian M.M. Vieira, Marco A. Minetto, Emma F. Hodson-Tole, Alberto Botter

**Affiliations:** a Escola de Educação Física e Desportos, Universidade Federal do Rio de Janeiro, Brazil; b Laboratorio di Ingegneria del Sistema Neuromuscolare (LISiN), Politecnico di Torino, Italy; c Division of Endocrinology, Diabetology and Metabolism, Department of Internal Medicine, University of Turin, Turin, Italy; d Institute for Biomedical Research into Human Movement and Health (IRM), Manchester Metropolitan University, UK

**Keywords:** Gastrocnemius muscles, Ankle torque, Electrical stimulation

## Abstract

Ankle movements in the frontal plane are less prominent though not less relevant than movements in the plantar or dorsal flexion direction. Walking on uneven terrains and standing on narrow stances are examples of circumstances likely imposing marked demands on the ankle medio-lateral stabilization. Following our previous evidence associating lateral bodily sways in quiet standing to activation of the medial gastrocnemius (MG) muscle, in this study we ask: how large is the MG contribution to ankle torque in the frontal plane? By arranging stimulation electrodes in a selective configuration, current pulses were applied primarily to the MG nerve branch of ten subjects. The contribution of populations of MG motor units of progressively smaller recruitment threshold to ankle torque was evaluated by increasing the stimulation amplitude by fixed amounts. From smallest intensities (12–32 mA) leading to the firstly observable MG twitches in force-plate recordings, current pulses reached intensities (56–90 mA) below which twitches in other muscles could not be observed from the skin. Key results showed a substantial MG torque contribution tending to rotate upward the foot medial aspect (ankle inversion). Nerve stimulation further revealed a linear relationship between the peak torque of ankle plantar flexion and inversion, across participants (Pearson *R* > .81, *p* < .01). Specifically, regardless of the current intensity applied, the peak torque of ankle inversion amounted to about 13% of plantar flexion peak torque. Physiologically, these results provide experimental evidence that MG activation may contribute to stabilize the body in the frontal plane, especially under situations of challenged stability.

## 1 Introduction

Movements of the foot are often conceived as occurring predominantly in the sagittal plane; these movements are termed dorsal flexion and plantar flexion. Less prominent, though not less relevant, lateral foot movements are equally frequently observed with respect to those occurring in the sagittal plane (e.g., when walking on uneven terrain, taking a side step or shifting weight laterally). Inappropriate control of ankle stability in the frontal plane, may explain the high incidence of lateral ankle sprains (Fong, Hong, Chan, Yung, & Chan, 2007). Lateral ankle motion might occur in the frontal and transverse planes, though both the talocrural and subtalar joints seem more predisposed to movements in the frontal plane (Sheehan, 2010). Given the relatively longer than wider feet dimension, lateral stabilization of the body possibly demands marked control of ankle movement in the frontal plane under circumstances of challenged stability (e.g., stance phase of gait; Cappellini, Ivanenko, Dominici, Poppele, & Lacquaniti, 2010; Henry, Fung, & Horak, 1998). Specific quantifications on the active contribution of leg muscles to ankle torques in the frontal plane, however, are currently unknown.

The pinnate gastrocnemius muscles are possible candidates for the active control of ankle lateral motion (Giordano, Segal, & Abelew, 2009; Lee & Piazza, 2008). Due to the strong relationship between ankle plantar-dorsal flexion and activation of gastrocnemius, these muscles are ubiquitously regarded as ankle extensors. Studies on muscle architecture and activation, nevertheless, suggest a potentially large contribution of these muscles to lateral ankle torques. For instance, the medial (MG) and lateral gastrocnemius attach obliquely to the Achilles tendon (Blitz & Eliot, 2007). Additionally, *in-vivo* estimates indicate significant values of gastrocnemius moment arms in the frontal plane (Lee & Piazza, 2008). Direct evidence from the cat gastrocnemius, indeed, supports the active production of torque outside the sagittal plane (Carrasco, Lawrence, & English, 1999; Lawrence, Nichols, & English, 1993). In humans, active torques in the frontal plane have been inferred from surface electromyograms (EMGs) detected in response to cutaneous reflexes (Zehr, Stein, & Komiyama, 1998), to postural sways occurring in quiet standing (Vieira, Windhorst, & Merletti, 2010) and to surface perturbations applied to subjects during static (Torres-Oviedo & Ting, 2007), as well as during more dynamic conditions, such as walking (Nieuwenhuijzen & Duysens, 2007) and upon landing impacts (Grüneberg, Nieuwenhuijzen, & Duysens, 2003). It seems, therefore, of relevance to estimate the amount of active ankle torque produced in the frontal and sagittal planes. Previously, we used a grid of electrodes to investigate the synchronization of postural EMGs in the gastrocnemius muscles (Vieira, Windhorst, et al., 2010). On occasions, we observed that both gastrocnemius heads were activated synchronously across individual forward sways. On other occasions, medial and lateral heads showed different activation timings. Strikingly, events of asynchronous activation between the two heads were associated to forward sway deviated laterally. This leads us to consider the controversial possibility that the human gastrocnemius muscle produces substantial ankle torque in the frontal plane, likely associated with the stabilization of lateral bodily sways.

In the current study, we used nerve electrical stimulation to test whether MG torques have significant components outside the sagittal plane. With a markedly small stimulation electrode, we aimed to stimulate the nerve branch serving exclusively the MG muscle (Wolf & Kim, 1997) and thus to quantify the electrically elicited twitches in different planes. Previously, Giordano et al. (2009) quantified the amplitude of plantar flexion and inversion moments produced by electrically elicited twitches in both gastrocnemius heads and in the fibularis longus muscle. By using different current intensities, in the current manuscript we further evaluate the relative contribution of different proportions of the whole population of MG motor units to frontal and sagittal torque components. To our knowledge, this is the first study to systematically report *in-vivo* contributions of MG motor units of different recruitment thresholds to ankle torque in both the frontal and sagittal planes.

In view of its documented moment arm of ankle inversion (∼4 mm; Lee & Piazza, 2008), we hypothesized that MG twitches would be significantly represented both in the sagittal (ankle plantar flexion) and frontal (ankle inversion) planes. If differences between frontal and sagittal MG torques are exclusively due to different moment arms in both planes, then, for a single joint position, we would not expect any relative variation in plantar flexion and inversion torques with stimulation intensity.

## 2 Methods

### 2.1 Subjects

Ten (one female) healthy subjects (range values: 23–36 years; 58–85 kg; 165–188 cm) participated in this study. Participants did not report musculo-skeletal dysfunctions and provided written informed consent before enrolling in the experiments. Experimental procedures conformed to the Declaration of Helsinki and were approved by the Institutional Ethical Committee of our university.

### 2.2 Experimental protocol

**Fig. 1:**
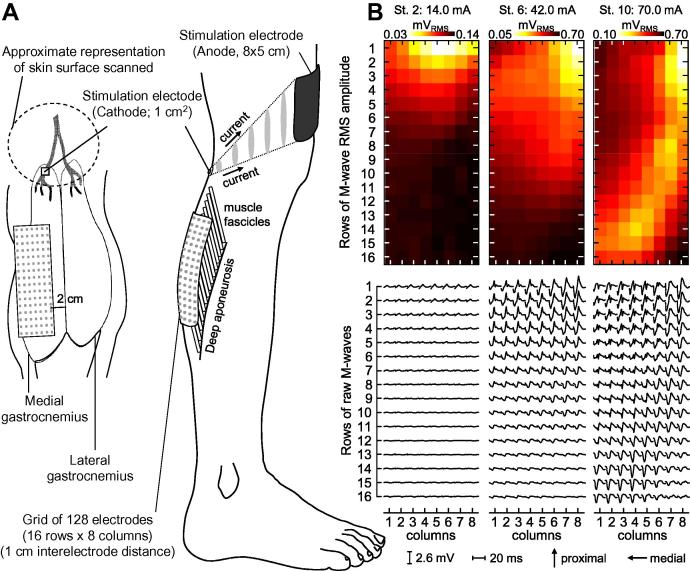
Positioning of stimulation and detection electrodes and M-waves. A, schematically shows the position of stimulation and detection electrodes in relation to the medial gastrocnemius (MG) muscle. After delivering current pulses over a relatively large region (dashed line) with a pen electrode, the pre-gelled cathode electrode was positioned at a specific location where the least injected current led to twitches observed primarily on skin regions closest to the MG muscle. The location of the stimulation electrode was presumably closest to the MG nerve branch. Arrows in the right panel indicate the direction along which electric charges flowed from the small cathode to the large anode electrode (monopolar stimulation modality; Merletti, Knaflitz, & DeLuca, 1992). Note that for a fixed current intensity, the amount of charges per unit of area (i.e., grey ellipses) reduces toward the anode electrode. Nerve fibers more distant from the cathode were therefore less sensible to variations in stimulation amplitude. B, shows raw M-waves (bottom panel) and their root mean square (RMS) amplitude obtained for the stimulation levels 2 (14 mA), 6 (42 mA) and 10 (70 mA) for the subject 2. Note the local representation of M-waves across the matrix. At stimulation level 2, small M-waves were predominantly represented at the more proximal rows. With the increase in stimulation amplitude large M-waves emerged laterally and distally.

The contribution of the MG muscle to inversion ankle torques was assessed while subjects were seated on a customized chair. With the ankle in neutral position, knee fully extended, trunk slightly (less than 10 °) extended and the hip joint flexed at angles ranging from 80 to 90 ° (0 ° means full hip extension), participants had their right foot pressed onto a force-plate positioned vertically in front of them (see Fig. 1 in Gallina, Merletti, & Vieira, 2011). Ankle neutral position was defined in terms of the average angle (∼88 °) observed between the foot and the tibia when participants stood uprights quietly. Such posture ensured the recording of ankle torque with the gastrocnemius muscles at lengths similar to those observed during standing.

Gastrocnemius twitches were elicited with monopolar electrical stimulation. When monopolar stimulation is used, the cathode and the anode stimulation electrodes are positioned respectively nearby and away the targeted nerve or motor point (Merletti, Knaflitz, & DeLuca, 1992; see Fig. 1A). In such a stimulation modality, the current density (i.e., amount of charge per unit of area) is markedly greater in proximity of the cathode electrode. Then, with a large anode electrode (80 × 50 mm, Spes Medica, Battipaglia, Italy) fixed immediately above the patella, bipolar current pulses (square wave: 200 μs duration) were selectively delivered to the tibial nerve with a small, pre-gelled stimulation electrode (cathode: 10 × 10 mm; Spes Medica, Battipaglia, Italy). According to anatomical reports on cadavers, there is a distinct primary branch of the tibial nerve serving the MG muscle. Regardless of how proximally this ramification occurs, it seems to occur prior to the nerve entry into the MG muscle (Parratte, Tatu, Vuillier, Diop, & Monnier, 2002; Wolf & Kim, 1997). Before positioning the cathode electrode, therefore, the nerve branch serving exclusively the MG muscle was scanned on the skin (see dashed ellipse in Fig. 1A) with a pen stimulation electrode (1 cm^2^ surface; Globus Italia, Codognè, Italy). When positioning the pen electrode relatively proximally, mechanical twitches could be clearly observed and palpated over the whole calf. By shifting the pen electrode progressively distally, twitches were found to occur predominantly closer to the MG head location. The pre-gelled cathode electrode was thus positioned in the most distal skin location leading to twitches observed consistently in correspondence to the MG muscle for the least injected current.

**Fig. 2:**
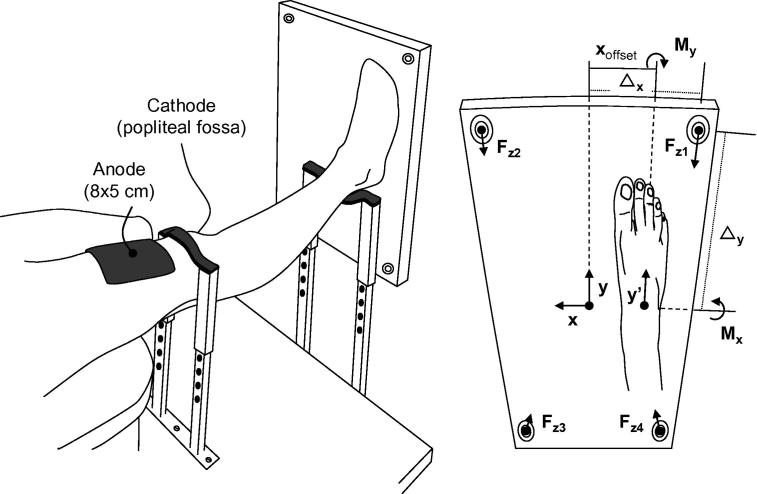
Ankle torque as calculated from reaction forces. A schematic representation of subjects positioning (left panel) and foot positioning (right panel) on the force-plate is shown. Indications on the location of the cathode (popliteal fossa) and anode (just proximally to the patella) electrodes are given. Note that cathode and anode were at respectively opposite dorsal and ventral body surfaces (i.e., monopolar stimulation modality). Rigid strips at knee and ankle levels were used to prevent knee bending during stimulation. As detailed in the right panel, the transversal axis of ankle rotation, about which the foot plantar-dorsal flexes, was aligned parallel along the force plate transversal axis (**x** axis). In this case, torque measured about the **x** axis corresponds to the ankle torque in the sagittal plane. The longitudinal axis (**y’**) of ankle inversion-eversion, although parallel to the force-plate **y** axis, was laterally shifted by a different amount (**X_offset_**) between subjects. Ankle torque in the sagittal (**M_x_**) and frontal (**M_y_**) plane was calculated from the reaction force (**F_z_**) measured by each of the four piezoelectric sensors located at the force-plate vertices (see text for equations).

Twitches of different magnitudes were elicited by varying the amplitude of current pulses. Stimulation pulses were delivered for 50 s, at a rate of 2 pps. Every ten stimuli (5 s), stimulation amplitude progressively increased by a fixed amount, from 10 to 100% of a predefined range. The first stimulation level corresponded to the smallest current for which the peak plantar flexion torque was significantly higher than the background noise level (i.e., two standard deviations higher). The last stimulation level matched the highest current intensity leading to greatest peaks in ankle twitches without any visible stimulation of other muscles. Given the selectivity provided by monopolar stimulation, MG nerve fibers were more likely elicited by increasing the current intensity than those serving other muscles. This concept is schematically illustrated in Fig. 1A; the amount of electric charge per unit of area is greater at regions closer to the cathode electrode. Moreover, rigid strips ensured knee bending did not occur during stimulation (Fig. 2). Indeed, even for the greatest stimulation amplitudes, leg or thigh movements were not observed.

### 2.3 Instrumentation

Ankle torque in the frontal and sagittal plane were calculated from reaction forces measured by highly sensitive sensors (∼0.9 mV/N; 9286AA Kistler, Zurich, Switzerland). Reaction forces were digitized using a 12 bit A/D converter, which provided an approximate resolution of ∼2.7 N/bit. Forces were recorded synchronously with surface EMGs at 2048 samples/s.

Surface EMGs were recorded to ensure that increases in stimulation amplitude elicited a progressively greater number of MG motor units. EMGs were collected with 128 circular electrodes (0.4 cm diameter), arranged into a grid of 16 rows × 8 columns with 1 cm center-to-center distance. The amplification factor (100–1,000) was set separately for each individual, to maximize signal-to-noise ratio without resulting in saturation (10–500 Hz EMG-USB amplifier, LISiN and OT Bioelettronica, Turin, Italy). The positioning of electrodes on skin regions located exclusively over the MG muscle was guided with ultrasound scanning (7.5 MHz linear-array transducer, MyLab^TM^ 25, Esaote, Genova, Italy). Considering the local representation of M-waves in surface EMGs collected from the pinnate gastrocnemius muscle (Hodson-Tole, Loram, & Vieira, 2012), we used ultrasound imaging to ensure most of the muscle volume was covered by our matrix of electrodes. Briefly, the junction between the two gastrocnemius heads and the distal extremity of MG superficial aponeurosis were scanned and marked on the skin; see the supplemental material in Vieira, Merletti, and Mesin (2010) for detailed indication on how to identify these anatomical locations in ultrasound images. After carefully cleaning the skin with alcohol, the eighth column of electrodes was positioned 2 cm medially from the gastrocnemius junction, whereas the bottom row of electrodes coincided with the distal extremity of MG aponeurosis. Through the use of such a careful procedure for electrode positioning, we ensured our grid of electrodes sampled M-waves regardless of whether they were represented in the more proximo-distal and/or medio-lateral MG regions (Fig. 1B).

### 2.4 Calculation of ankle torque

Ankle torque was estimated from reaction forces measured by a piezoelectric force-plate. Ankle plantar flexion torque was calculated as the torque **M_x_** about the force-plate **x** axis (Fig. 2), after assuring it was as parallel as possible to the line connecting the tips of medial and lateral malleolus (i.e., parallel to the ankle transverse axis; Wu et al., 2002). Parallelism was conceived in terms of markedly small **M_x_** values (smaller than 0.1 Nm) when subjects had their foot passively pressing on the force-plate surface. Plantar flexion was defined as positive **M_x_** values:

(1) $$M_{x}=(F_{z1}+F_{z2}-F_{z3}-F_{z4})\Delta_{y}$$

where **F_z_** correspond to the perpendicular reaction forces and Δ**_y_** is the distance (0.25 m) between each sensor and the **x** axis. Ankle inversion-eversion torque, instead, was calculated with respect to an axis parallel to the **y** axis (Fig. 2):

(2) $$M_{y}=(F_{z2}+F_{z3})(\Delta_{x}+x_{\mathrm{offset}})-(F_{z1}+F_{z4})(\Delta_{x}-x_{\mathrm{offset}})$$

where Δ**_x_** is the distance (0.125 m) between sensors and **y** axis. **x_offset_** corresponds to the distance between **y’** axis (Wu et al., 2002), roughly defined as the line connecting the calcaneus tuberosity to the third metatarsal head, and **y** axis. Positive **M_y_** values indicate inversion torques.

### 2.5 Data analysis

Representative twitches were obtained by averaging MG responses across ten stimuli. Initially, force data were low-pass filtered with a second order Butterworth filter (10 Hz, cut-off frequency). Then, trigger signals coding stimulation onsets were used to split the time series of ankle torque into ten epochs (500 ms). After that, for each stimulation amplitude, the peak torque of ankle eversion-inversion and plantar-dorsal flexion was computed by getting the absolute maximal **M_y_** and **M_x_** values.

The degree of MG stimulation was estimated from EMG (M-wave) amplitude. After being filtered from 15 to 350 Hz (second order Butterworth filter), surface EMGs were split into 500 ms epochs. Root mean square (RMS) values were then calculated for individual epochs of 20 ms centered over each M-wave; the spatial distribution of M-wave amplitude was calculated across the grid of electrodes (Fig. 1B). Considering the amplitude of M-waves distributes unevenly across the gastrocnemius volume (Fig. 1B; see also Hodson-Tole et al., 2012), RMS values were averaged across stimuli and M-waves to provide a representative index of global MG activation for each stimulation intensity:

(3) $$\text{RMS}[\mathrm{ci}]=\frac{1}{N_{s}\mathrm{ch}}\overset{N_{s}}{\underset{s=1}{\sum }}\overset{128}{\underset{\mathrm{ch}=1}{\sum }}\sqrt{\frac{1}{41}\overset{41}{\underset{i=1}{\sum }}\mathrm{EMG}{\left[\mathrm{ch}\text{,}i\Delta t+t_{s}\right]}^{2}}$$

where *EMG*[*ch*,*iΔt*+*t_s_*], Δ*t* and *i* correspond respectively to the raw EMGs recorded with the channel number *ch*, the sampling interval (1/2048 sec) and an integer number ranging from 1 to 41 (41 samples amount to ∼20 ms). *t_s_* are the instants corresponding to the delivery of stimulation pulses, with *s* ranging from 1 to the total number of stimuli (*N_s_* = 10) applied for each current intensity (*ci*).

### 2.6 Statistical analysis

Correlation analyses were considered to investigate the association between: (i) MG activation and twitch amplitude and (ii) MG inversion and plantar flexion torques. Pearson correlation coefficient was calculated to ascertain that greater ankle torque resulted from stronger MG stimulation. Changes in the torque of ankle inversion were tested with the non-parametric Friedman ANOVA, with stimulation amplitudes as repeated measures. Finally, regression analysis was applied to quantify the relationship between plantar flexion and inversion torques.

## 3 Results

### 3.1 Medial gastrocnemius activation and twitch torque

**Fig. 3:**
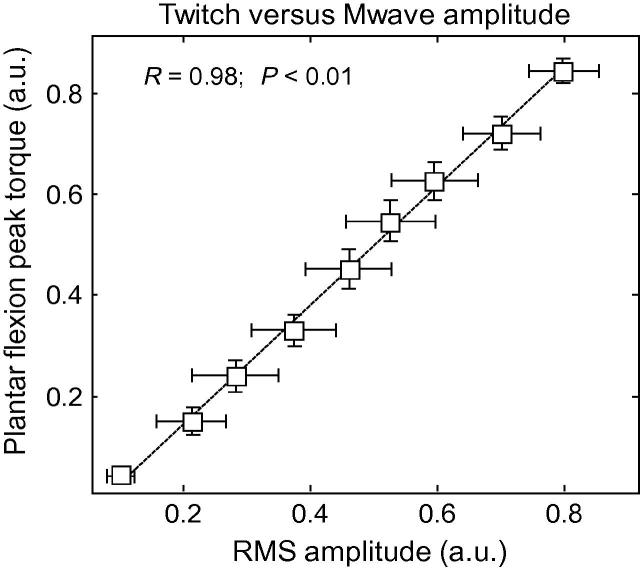
Increases in muscle activation and twitch. The peak torque of plantar flexion produced by stimulation of the MG nerve branch is plotted against the root mean square amplitude of M-waves. Values were normalized with respect to those obtained for the maximal stimulation intensity and averaged (whiskers indicate standard deviation) across subjects and across the nine preceding stimulation intensities, from 10% to 90% of the maximal. Note that increases in the amplitude of MG twitches were consistently and strongly associated to increases in M-wave amplitude (see Pearson Coefficient of Correlation and the significance level). The dashed trace represents the regression line fitted to the data.

Plantar flexion torque and M-wave amplitude were strongly associated. The greater the M-wave RMS amplitude, the larger the plantar flexion torque (Fig. 3). Despite the relatively large variation in overall RMS amplitude (see whiskers in Fig. 3), the degree of MG stimulation was significantly associated to peak plantar flexion torques (Pearson *R* = .98, *p* < .01).

### 3.2 Electrically elicited inversion torque: representative result

**Fig. 4:**
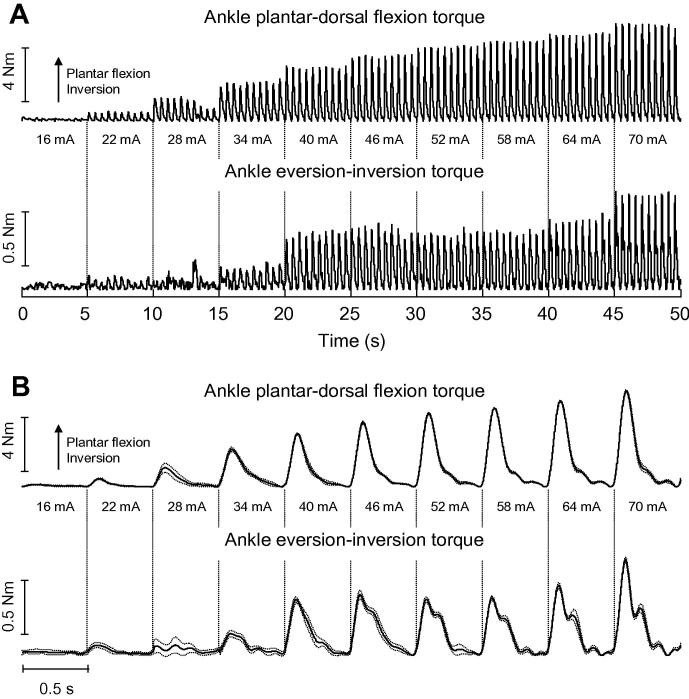
Medial gastrocnemius twitches. A, shows the twitches produced by subject 10 when the nerve branch serving his MG muscle was stimulated with 100 bipolar current pulses. Stimulation amplitude increased progressively for every set of ten stimuli, starting from 10% (16 mA) of the maximal stimulation amplitude (70 mA; see text for the definition of maximal stimulation amplitude). Twitches were calculated separately for the sagittal (top; positive values indicate plantar flexion) and frontal planes (bottom; positive values indicate inversion torque). Gastrocnemius twitches averaged across stimuli with same intensity are shown in B. Dashed lines correspond to the standard deviation of twitches amplitude. Note that, although at different extents, plantar flexion and inversion twitches get progressively bigger with the increase in stimulation amplitude.

Stimulation of the tibial nerve led to mechanical twitches represented in the sagittal and frontal planes. As the intensity of current pulses augmented, MG torque in both planes increased progressively. Fig. 4 shows representative twitches obtained for Subject 10. At the smallest intensity, stimulation pulses resulted in negligible plantar flexion and inversion torque (Fig. 4). When stimulated at higher intensities, however, plantar flexion and inversion torques increased consistently. Apart from the third stimulation level (28 mA), twitch amplitude showed remarkably small variation (compare dotted and solid lines in Fig. 4B). Notwithstanding their absolute values, substantial active torques were evident in and outside the sagittal plane.

### 3.3 Electrically elicited inversion torque: group results

**Fig. 5:**
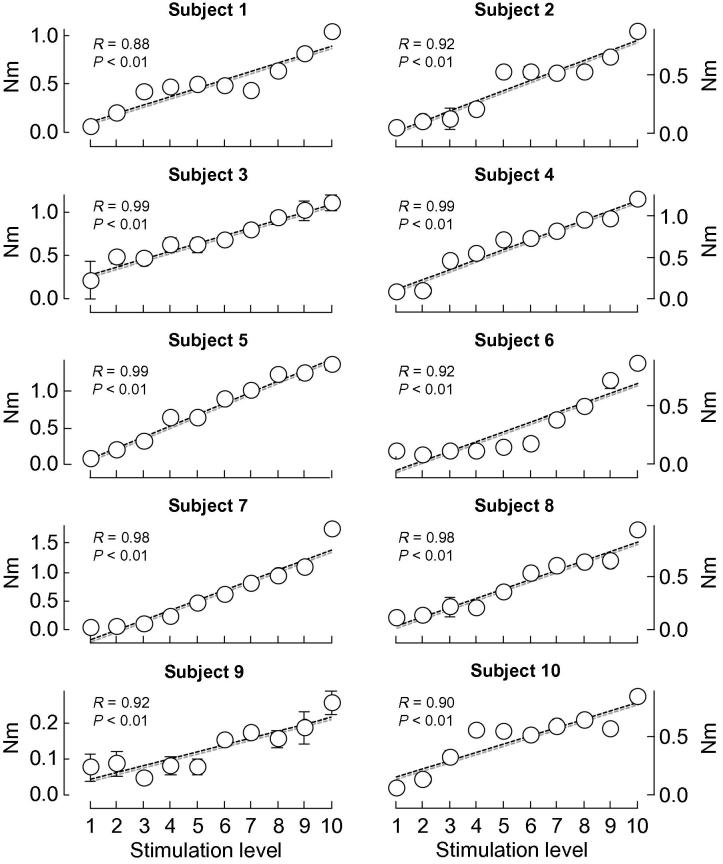
Gastrocnemius peak torque of ankle inversion. Peak torques of ankle inversion, produced by stimulation of the MG nerve branch, are shown for each of the twelve subjects tested. Torque values were averaged over individual sets of stimulation pulses, with each set comprising ten stimuli of equal intensity level. Ten stimulation levels were tested, with the first and the last levels corresponding, respectively, to 10% and 100% of the maximal stimulation intensity. Whiskers, whenever present, indicate standard deviation values. From the first to the last stimulation level, the torque of ankle inversion increased consistently across subjects (see Pearson *R* coefficients and their significance level). Dashed traces represent regression lines fitted to individual scatter plots.

All participants exhibited significant inversion torques during stimulation. Regardless of the stimulation amplitude, torque values were remarkably consistent across stimuli and participants (median coefficient of variation: 8.0%; *N* = 100 cases). Statistical analysis revealed a significant additive effect of stimulation intensity on the amplitude of inversion torque (Friedman ANOVA; *N* = 12, *p* < .001). In fact, increases in inversion torque were significantly associated with increases in current intensity (Fig. 5; Pearson *R* > .88 and *p* < .01 in all cases). Inspection of Fig. 5, however, reveals that not all subjects showed similar torque-stimulation profiles. In some subjects, inversion torque increased linearly with stimulation amplitude. Other Subjects showed short plateaus in peak inversion torque (subjects 2, 6, 9 and 10).

### 3.4 Linear relationship between inversion and plantar flexion torques

**Fig. 6:**
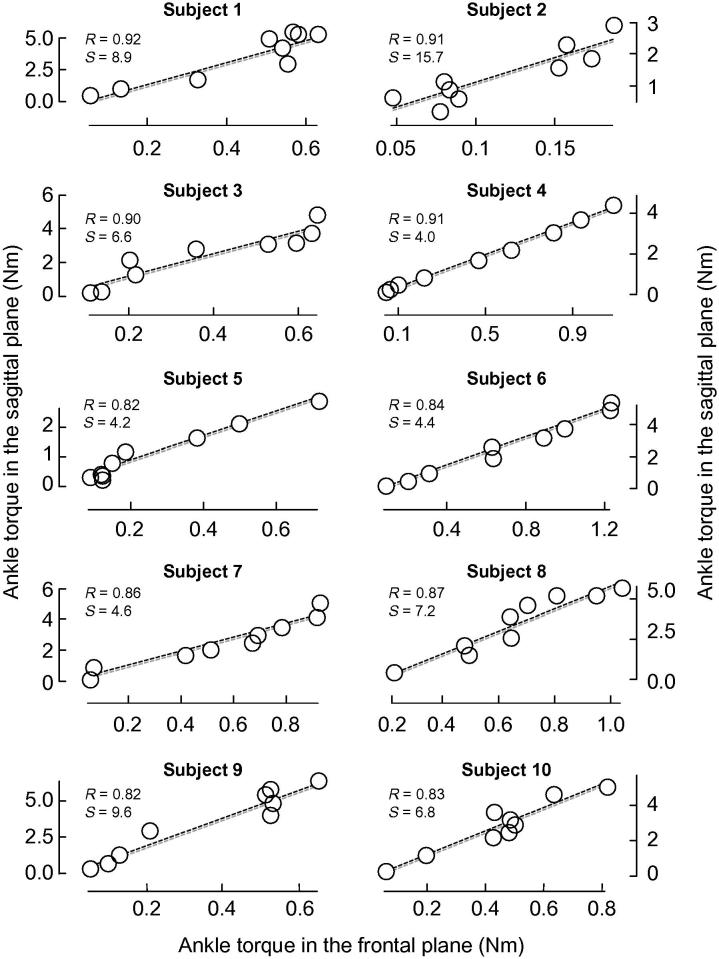
Ankle plantar flexion versus ankle inversion peak torques. The scatter plot shows MG peak torques in different planes and for each participant separately. Each circle denotes the peak torque of ankle plantar flexion (ordinate) and ankle inversion (abscissa), averaged across ten stimuli for each stimulation level and participant. Pearson Correlation Coefficient (*R*) and the slope (*S*) of regression lines (dashed line) fitted to individual data are shown. In all cases the correlation coefficient reached statistical significance (*P* < .01). Note the strong linear association between torques of plantar flexion and ankle inversion, with the torque of ankle inversion corresponding to about 13% of the plantar flexion torque on average.

Increases in plantar flexion torque were accompanied by a proportionally fixed increment in inversion torque. Considering the significant linear correlation between plantar flexion and inversion torques for all participants (Pearson *R* > .81, *p* < .01), the relative contribution of frontal and sagittal MG torque to the total ankle torque was estimated with linear regression (Fig. 6). On average, the lowest current pulses resulted in relatively small ankle torques in both planes. With the progressive increase in stimulation amplitude, however, plantar flexion and inversion torque increased at a different, though proportional rate. For every 1 Nm increase in plantar flexion torque there was on average a corresponding ∼0.13 Nm (range: 0.06–0.24 Nm) increase in inversion torque (see individual slope values shown in Fig. 6).

## 4 Discussion

### 4.1 How selectively did current pulses stimulate the medial gastrocnemius?

With a small adhesive electrode, we aimed to stimulate the tibial nerve branch exclusively supplying the MG muscle. Although the possibility of having stimulated other plantar flexors cannot be excluded based on our data, the careful procedure we considered to position the stimulation electrodes in this study (Fig. 1B) presumably ensured the current pulses stimulated primarily the MG motoneurons. Twitches elicited by small current intensities were, thus, very likely associated to exclusive MG stimulation. By augmenting the current intensity, though, the MG contribution to ankle torque and the likelihood of stimulating other muscles increased. Two specific procedures were however further considered to circumvent the stimulation of other muscles. First, current pulses were delivered in monopolar modality. In such stimulation modality, greater amounts of electrical charges are selectively delivered to nerve fibers closer to the cathode electrode (Fig. 1A; see also Merletti et al., 1992). Second, the maximal stimulation amplitude was kept relatively low. Indeed, the values of maximal peak torque of ankle plantar flexion observed here (5.4 ± 1.5 Nm) were less than half of those commonly reported in the literature (∼12 Nm) for supramaximal nerve and muscle stimulation (Sale, Quinlan, Marsh, McComas, & Belanger, 1982; van Zandwijk, Bobbert, Harlaar, & Hof, 1998).

It might be argued that surface electrodes placed over the lateral gastrocnemius and soleus muscles could have indicated whether current pulses had exclusively stimulated the MG muscle. We, however, focused on using a large grid of electrodes to sample M-waves across the whole MG volume (Fig. 1B); M-waves are unlikely appreciated in surface EMGs collected from a specific MG region. Reports on the inhomogeneous distribution of activity within the gastrocnemius muscles are emerging in the literature (Kinugasa, Kawakami, & Fukunaga, 2005; Staudenmann, Kingma, Daffertshofer, Stegeman, & van Dieën, 2009; Wolf, Segal, & English, 1992). Recently, we showed that because of its pinnate architecture, motor unit action potentials in the MG muscle are represented locally on the skin (Vieira, Loram, Muceli, Merletti, & Farina, 2011). Had we used conventional recording systems, based on the sampling of muscle activity with a single bipolar recording, the stimulation of MG units residing in muscle regions not sampled by the surface electrodes would not be represented in the surface EMGs (Mesin, Merletti, & Vieira, 2011; see Fig. 1 in Hodson-Tole et al., 2012). We therefore used a large matrix of electrodes to ensure that changes in ankle torque observed with increases in stimulation amplitude were, indeed, associated with the stimulation of populations of MG motor units (Fig. 3). Because of their pinnate architecture, obtaining representative M-waves from other calf muscles would require a similarly large detection system. It should be noted that, even if surface electrodes had been placed on skin regions covering other muscles, it would not suffice to ascertain whether current pulses had stimulated the soleus muscle. In view of the markedly high selectiveness of surface recordings from pinnate muscles (Mesin et al., 2011; Vieira et al., 2011), and given that the medio-proximal soleus region is covered by the gastrocnemius muscle, ensuring no soleus motor units were stimulated would require the use of several arrays of intramuscular electrodes positioned at different soleus regions. Currently available means, then, would not allow for ascertaining how strongly other calf muscles contributed to ankle torque.

### 4.2 What are the origins and consequences of gastrocnemius inversion torque?

The triceps surae muscles are commonly conceived as ankle extensors. While specific studies reported frontal and horizontal torques for the cat gastrocnemius (Carrasco et al., 1999; Lawrence et al., 1993), in humans, similar quantifications were recently studied during electrically elicited contractions at constant stimulation amplitudes (Giordano et al., 2009). Perhaps because of the predominant ankle movement in the sagittal plane, active contribution of individual calf muscles to ankle torques outside the sagittal plane is often disregarded. Current results (Fig. 5), however, indicate a significant active MG contribution to ankle inversion.

Notwithstanding the complex anatomy of the ankle joint, the inversion torques elicited electrically in this study likely resulted from an inversion moment arm of the MG muscle. Barnett and Napier (1952), for example, observed different curvatures in the lateral and medial aspects of the talus trochlear surface, which depended on the ankle position in the sagittal plane. During plantar flexion, these authors observed a downward and medial inclination of the talocural rotation axis. Therefore, if the MG torques were directed entirely towards plantar flexion, MG activation would possibly result in ankle eversion rather than inversion. Inversion torques observed here, then, were accounted for by other mechanisms. Indeed, *in vivo* estimates indicate a significant inversion moment arm (∼4 mm) for the human MG muscle with the foot in neutral position (Lee & Piazza, 2008). This average value, however, is about nine times smaller than those estimated for the plantar flexion moment arm (∼35 mm), even though these estimates were obtained for the Achilles tendon rather than for the MG-tendon junction (Fath, Blazevich, Waugh, Miller, & Korff, 2010). Such average difference in plantar flexion and inversion moment arms appears to predict well the ratio between MG twitches in sagittal and frontal planes (Fig. 6).

The relatively large MG lever-arm in the frontal plane may have relevant functional consequences. For instance, when subjects walk along either stable or unstable surfaces, the gastrocnemius muscle could be actively engaged in the lateral stabilization of their body center of mass over a small support base (Nieuwenhuijzen & Duysens, 2007). This observation seems, indeed, supported by early MG activation and co-activation of ankle muscles observed during the stance phase for subjects walking on slippery surfaces (Cappellini et al., 2010). A similar pattern of MG activation was additionally reported for patients with peripheral neuropathy (Kwon, Minor, Maluf, & Mueller, 2003), further evidencing the MG role in lateral foot stabilization. On the other hand, given that MG inversion torque amounts to ∼13% of its plantar flexion torque (Fig. 6), disproportionate MG strengthening with respect to other ankle muscles could lead to marked differences in ankle eversion-inversion torques. The uneven distribution of strength between ankle muscles seems, indeed, one of several possible risk factors accounting for lateral ankle sprains (Baumhauer, Alosa, Renström, Trevino, & Beynnon, 1995; Beynnon, Murphy, & Alosa, 2002; Fong et al., 2007). Therefore, reinforcing the MG muscle in addition to other ankle inversors seems advisable for ensuring lateral ankle stability.

### 4.3 Possible reasons for non-linear relationships between inversion torque and stimulation intensity

Although inversion torque increased with stimulation amplitude, such increase was not always linearly related to stimulation amplitude. Non-linear associations were evident for the subjects 2, 6, 9 and 11 shown in Fig. 5. When increasing the stimulation amplitude, a new population of motor units might be stimulated (Fig. 1B). The number and the size of motor units stimulated will depend on their recruitment threshold (i.e., on the diameter of their nerve fibers) in relation to the stimulus intensity. It is therefore possible that a small number of, or none of, the motor units in the gastrocnemius muscle has been stimulated when stimulation amplitude increased through consecutive levels (Botter, Merletti, & Minetto, 2009). It is also possible that motor units recruited for a certain stimulation level contribute more strongly to ankle torque. For these reasons, and possibly due to the stimulation of other muscles for the greatest current intensities, changes in ankle torque and stimulation amplitude are not necessarily linearly related.

### 4.4 Physiological implications for the linear relationship between plantar flexion and inversion torques

Depending on the attachment site of active fibers in relation to the muscle tendon, individual skeletal muscles might produce force in different directions (Desmedt & Godaux, 1981; ter Haar Romeny, van der Gon, & Gielen, 1984). The directional tuning of muscle force seems to depend more directly upon the location of the territory of motor unit within the muscle volume rather than on the degree of muscle activation or on whether muscle fibers attach to broad bony regions or converge towards strap-like tendons (Herrmann & Flanders, 1998). Whether a motor unit is recruited in the first dorsal interosseus muscle, for example, appears to depend on the requested flexion-abduction force direction (Desmedt & Godaux, 1981). Similar force-direction specificity has been reported for motor units in other muscles (Riek & Bawa, 1992; ter Haar Romeny et al., 1984). It seems then pertinent to ask whether the preferential direction of ankle torque changes with the stimulation of MG motor units of different recruitment thresholds. If this is the case, either plantar flexion or inversion torque would increase more strongly for greater stimulation levels.

Changes in ankle torque observed for increases in stimulation intensity were likely related to the size of MG motor units (i.e., to the diameter of MG nerve fibers). When electrical stimuli are delivered to the muscle motor point, the motor units elicited depend both on the diameter and on the location of their nerve fibers (Feiereisen, Duchateau, & Hainaut, 1997). During nerve stimulation, on the other hand, nerve fibers are progressively recruited according to their size. Larger axons, in virtue of their lower electrical threshold, are more highly excitable than smaller axons to current fields imposed externally. On this view, the variation in plantar flexion and inversion torques reported here were, presumably, due to the stimulation of progressively smaller motor units. In this case, and assuming current pulses were predominantly delivered to the MG nerve branch (Fig. 1A), the linear relationship between peak torques in sagittal and frontal planes (Pearson *R* > .81; Fig. 6) suggests that MG motor units of different recruitment thresholds contributed proportionally equally to ankle torques in both planes. This finding is consistent with the non-significant association observed between the recruitment threshold of motor units in the biceps brachialis and the force direction along which these units were preferentially recruited (Herrmann & Flanders, 1998). Here, we are not claiming that MG motor units produce a fixed proportion of ankle torque in different directions. Our results, instead, indicate that any predominant contribution of MG motor units to a specific direction relies not upon their size but upon other mechanisms, such as the control of fibers residing in specific muscle regions (Riek & Bawa, 1992). Although recent evidence suggests an inhomogenous distribution of synaptic inputs across MG motoneurons (Staudenmann et al., 2009; Vieira, Windhorst, et al., 2010; Vieira, Loram, Muceli, Merletti, & Farina, 2012), further testing is required to ascertain whether directional tuning of MG units exists.

The linear relationship between plantar flexion and inversion ankle torques further suggests a potential MG role in the lateral stabilization of postural sways. Because of the markedly small values of active ankle torque observed during standing (∼5 Nm; Jacono, Casadio, Morasso, & Sanguineti, 2004), the motor units recruited in quiet standing likely correspond to the smallest MG units. If highest current intensities applied here sufficed to stimulate the smallest units, then, postural MG motor units might produce relative amounts of inversion/plantar flexion torques similar to those produced by larger units. Considering that ankle and hip movements are less associated for narrower stance widths (Day, Steiger, Thompson, & Marsden, 1993), inverting ankle torques produced by MG postural units would not be surprising. Indeed, surface EMGs indicate a consistent MG response to surface perturbations inducing ankle flexion-eversion movements (Henry et al., 1998). Similarly, changes in MG thickness with ageing were accompanied by abnormally higher lateral sways in older subjects only when standing over narrow stance (Onambele, Narici, & Maganaris, 2006). Finally, MG inversion torques could have additional implications for the arrest of lateral falls in the elderly. King and Horak (2008), for example, observed that Parkinsonians fall more often than controls in response to lateral surface translations. Interestingly, the likelihood of falling was not associated to the use of different stepping strategies. Patients were more prone to falling likely due to their delayed, shorter and slower lateral, stepping reaction. Whether or how much the MG muscle contributes to the compensation of lateral falls remains unknown. Our current results, however, suggest that MG contribution to ankle inversion torque amounts to 13% of its contribution to plantar flexion torque; collectively to the activation of other ankle inversors, MG activation might therefore contribute substantially to ankle lateral stabilization.

### 4.5 Possible limitations

It might be argued that eventual inter-segmental movements occurring while subjects were being stimulated could have affected the distribution of reaction forces and, thus, the shape and the amplitude of calculated MG twitches. During the experiments, though, if subjects moved in an attempt to avoid discomfort or as a consequence of stimulation pulses (e.g., knee bending) it was clearly not observable. It should also be noted that we kept the maximum stimulation amplitude relatively low, so as to avoid discomfort and to minimize the possibility of stimulating other muscles (e.g., quadriceps femoris; Fig. 1A). In addition, as we aligned the ankle transverse axis as parallel as possible to the force-plate transverse axis, any pushing on the force-plate resulting from inter-segmental adjustments would unlikely affect the calculated peaks of ankle torque. Foot movements, on the other hand, could have accounted for changes in the shape of inversion twitches with stimulation amplitude (Fig. 4B). Direct measures of combined ankle and foot stiffness in the literature were found in the sagittal (Loram & Lakie, 2002) but not in the frontal plane. Given the narrow foot dimension, it is possible that in response to stimulus of greater amplitudes, movements of the foot in the medio-lateral directions were more prominent than those in the anterior-posterior direction. However, from twitches shown in Fig. 4, these movements, if occurred, seem to have not affected the peak torque of ankle inversion. We therefore believe that torque increments elicited by greater current pulses (Figs. 4 and 5) were presumably due to the stimulation of a progressively larger population of MG motor units and/or of other muscles. The chief limitation of the current study is the fact that we are not able to discriminate how much the proportional plantar flexion/inversion increase in ankle torque with stimulation amplitude (Fig. 6) was a consequence of moment arm, ankle anatomy or foot misalignments. While these aspects remain the subject of future investigations, possibly combining electromyography and ultrasound imaging, here we show that when delivered to skin regions presumably closest to the MG nerve branch, current pulses elicit ankle torque represented in orthogonal directions.
